# Validation of the Alzheimer’s disease-resemblance atrophy index in classifying and predicting progression in Alzheimer’s disease

**DOI:** 10.3389/fnagi.2022.932125

**Published:** 2022-08-05

**Authors:** Qiling He, Lin Shi, Yishan Luo, Chao Wan, Ian B. Malone, Vincent C. T. Mok, James H. Cole, Melis Anatürk

**Affiliations:** ^1^UCL Queen Square Institute of Neurology, Faculty of Brain Sciences, University College London, London, United Kingdom; ^2^Department of Imaging and Interventional Radiology, Faculty of Medicine, The Chinese University of Hong Kong, Hong Kong, Hong Kong SAR, China; ^3^BrainNow Research Institute, Shenzhen, China; ^4^School of Biomedical Sciences, Faculty of Medicine, The Chinese University of Hong Kong, Hong Kong, Hong Kong SAR, China; ^5^Dementia Research Centre, Department of Neurodegenerative Disease, UCL Queen Square Institute of Neurology, Faculty of Brain Sciences, University College London, London, United Kingdom; ^6^Gerald Choa Neuroscience Centre, Therese Pei Fong Chow Research Centre for Prevention of Dementia, Lui Che Woo Institute of Innovative Medicine, Division of Neurology, Department of Medicine and Therapeutics, Prince of Wales Hospital, Faculty of Medicine, The Chinese University of Hong Kong, Hong Kong, Hong Kong SAR, China; ^7^Department of Computer Science, Faculty of Engineering Science, University College London, London, United Kingdom; ^8^Dementia Research Centre, Institute of Neurology, University College London, London, United Kingdom; ^9^Department of Psychiatry, University of Oxford, Oxford, United Kingdom

**Keywords:** Alzheimer’s disease, Alzheimer’s disease-resemblance atrophy index, Minimal Interval Resonance Imaging in Alzheimer’s Disease, AD diagnosis, AD progression prediction, linear mixed-effects modelling, repeatability, reproducibility

## Abstract

**Background:**

Automated tools for characterising dementia risk have the potential to aid in the diagnosis, prognosis, and treatment of Alzheimer’s disease (AD). Here, we examined a novel machine learning-based brain atrophy marker, the AD-resemblance atrophy index (AD-RAI), to assess its test-retest reliability and further validate its use in disease classification and prediction.

**Methods:**

Age- and sex-matched 44 probable AD (Age: 69.13 ± 7.13; MMSE: 27–30) and 22 non-demented control (Age: 69.38 ± 7.21; MMSE: 27–30) participants were obtained from the Minimal Interval Resonance Imaging in Alzheimer’s Disease (MIRIAD) dataset. Serial T1-weighted images (*n* = 678) from up to nine time points over a 2-year period, including 179 pairs of back-to-back scans acquired on same participants on the same day and 40 pairs of scans acquired at 2-week intervals were included. All images were automatically processed with AccuBrain^®^ to calculate the AD-RAI. Its same-day repeatability and 2-week reproducibility were first assessed. The discriminative performance of AD-RAI was evaluated using the receiver operating characteristic curve, where DeLong’s test was used to evaluate its performance against quantitative medial temporal lobe atrophy (QMTA) and hippocampal volume adjusted by intracranial volume (ICV)-proportions and ICV-residuals methods, respectively (HVR and HRV). Linear mixed-effects modelling was used to investigate longitudinal trajectories of AD-RAI and baseline AD-RAI prediction of cognitive decline. Finally, the longitudinal associations between AD-RAI and MMSE scores were assessed.

**Results:**

AD-RAI had excellent same-day repeatability and excellent 2-week reproducibility. AD-RAI’s AUC (99.8%; 95%CI = [99.3%, 100%]) was equivalent to that of QMTA (96.8%; 95%CI = [92.9%, 100%]), and better than that of HVR (86.8%; 95%CI = [78.2%, 95.4%]) or HRV (90.3%; 95%CI = [83.0%, 97.6%]). While baseline AD-RAI was significantly higher in the AD group, it did not show detectable changes over 2 years. Baseline AD-RAI was negatively associated with MMSE scores and the rate of the change in MMSE scores over time. A negative longitudinal association was also found between AD-RAI values and the MMSE scores among AD patients.

**Conclusions:**

The AD-RAI represents a potential biomarker that may support AD diagnosis and be used to predict the rate of future cognitive decline in AD patients.

## Introduction

Alzheimer’s disease (AD) is a neurodegenerative disease progressively leading to dementia characterised by memory loss, confusion, mood changes and difficulties in communicating, problem-solving or coordination (Pini et al., 2016; Knopman et al., 2021; Leng and Edison, 2021; Scheltens et al., 2021). While treatments for AD are scarce, preclinical/prodromal biomarkers (e.g., amyloid β, pathological tau protein, brain atrophy) hold promise for the earlier disease detection and the development of novel interventions designed to prevent or delay AD (Dubois et al., 2014, 2021; Jack et al., 2018; Srivastava et al., 2021; Mahaman et al., 2022; Teunissen et al., 2022). For example, atrophy of the medial temporal lobe and hippocampus, as measured using structural magnetic resonance imaging (MRI), are now widely used AD biomarkers (Jack et al., 2011; Clerx et al., 2013).

The AD resemblance atrophy index (AD-RAI) is a novel machine-learning based brain atrophy biomarker for AD diagnosis (Zhao et al., 2019; Liu et al., 2021; Mai et al., 2021, 2022). Unlike single brain regional biomarkers, the AD-RAI summarises atrophy across multiple brain regions known to be affected by AD including subcortical structures, ventricles, and cortical lobar regions. This composite marker of AD-related brain atrophy ranges from 0 to 1, where a higher AD-RAI score indicates a greater amount of whole-brain atrophy matching the typical progression patterns in AD (Zhao et al., 2019; Liu et al., 2021; Mai et al., 2021, 2022). The AD-RAI has previously been linked to greater cognitive decline over 2 years and represents a strong predictor of conversion to mild cognitive impairment (MCI) and AD with dementia in cognitively unimpaired (CU) participants and MCI patients, respectively (Zhao et al., 2019). The AD-RAI has also outperformed single brain regional atrophy measurements (e.g., hippocampus, medial temporal lobe, bilateral temporal lobe) in the discriminating between AD patients and normal controls (Mai et al., 2021), in detecting early AD at prodromal stage (MCI due to AD) (Liu et al., 2021), and in predicting disease progression (Zhao et al., 2019). Most recently, the AD-RAI was demonstrated to predict the progression to AD in MCI patients carrying the APOE ε4 allele (Koutsodendris et al., 2022; Mai et al., 2022).

The potential clinical utility of the AD-RAI in facilitating earlier AD diagnosis and identifying at-risk individual AD warrants further exploration. As obtaining large numbers of AD patients is practically difficult for clinical study, the sample sizes of previous studies – especially those of a longitudinal nature – have been relatively small. This has limited the extent to which the AD-RAI has been assessed for technical reliability, repeatability, and reproducibility (Bartlett and Frost, 2008), although a small analysis of 11 participants (Mai et al., 2021) has previously found that AD-RAI scores generated from 1.5T to 3T MRI scanners had strong inter-scanner agreement when classifying individuals into patients or controls. Besides inter-scanner reproducibility, it is yet to be determined whether the AD-RAI has good repeatability (i.e., a high correspondence between AD-RAI scores taken from the same participant under identical conditions) and good reproducibility under other changing conditions other than pathological atrophy (such as a high correspondence between AD-RAI scores taken from the same participant over a period of time) (Bartlett and Frost, 2008). Furthermore, although the group-level association between AD-RAI and cognition decline has been revealed (Zhao et al., 2019), it remains unclear as to whether the AD-RAI is able to accurately capture longitudinal changes in cognitive impairment at individual level. With the aim to examine the same-day repeatability and 2-week reproducibility of the AD-RAI and further validate its use in disease classification and prediction, in a longitudinal setting, we selected the Minimal Interval Resonance Imaging in Alzheimer’s Disease (MIRIAD) database for our study. The MIRIAD dataset has stringently controlled scan and re-scan study design with a wide range of inter-scan intervals over 2 years, allowing formal assessments of the bias, repeatability and reproducibility of measures of atrophy (Malone et al., 2013).

## Materials and methods

### Participants

Data were obtained from the MIRIAD database through the MIRIAD XNAT database. Details of the study and sample characteristics can be found in Malone et al. (2013). In brief, the original MIRIAD project recruited 46 patients with probable AD (aged 55+) and 23 age-matched, non-demented controls. AD patients were diagnosed using the National Institute of Neurological and Communicative Disorders and Stroke and the Alzheimer’s Disease and Related Disorders Association (NINCDS-ADRDA) criteria (McKhann et al., 1984) and were required to have a Mini-Mental State Examination (MMSE) score (Folstein et al., 1975) between 12 and 26 to be eligible for the study. On the other hand, control participants were only included if they had a MMSE between 27 and 30 and did not have a history of cognitive impairment, head injury, major psychiatric disease or stroke.

### Image processing and Alzheimer’s disease-resemblance atrophy index calculation

Details of the image acquisition and pre-processing pipeline are available in the Supplementary Methods or in Malone et al. (2013) and in Abrigo et al. (2019). In brief, serial T1-weighted images were acquired on the participants at up to 9-time points over a 2-year period. For most of the participants, two back-to-back scans were also acquired within 1 day at 3-time points. The images collected using a 1.5T MRI scanner were automatically processed using AccuBrain^®^ IV1.2 system (BrainNow Medical Technology Limited). Volumetric measures [volume ratios of subcortical regions/ventricle structures to the intracranial volume (ICV); ratios of the CSF volume to the cortical volume of specific regions] were extracted for each participant. These measures were used to compute the AD-RAI, a single atrophy index ranging from 0 to 1, where higher scores indicates greater AD-like brain atrophy.

To compare the performance of the AD-RAI in AD diagnosis with established single-region biomarkers, quantitative medial temporal lobe atrophy (QMTA), hippocampal volume ratio (HVR), and hippocampal residual volume (HRV) were either obtained automatically through AccuBrain^®^ or computed manually. The QMTA was the ratio of the inferior lateral ventricle to the ipsilateral hippocampus volume and the HVR was defined as the ratio of the bilateral hippocampal volume (HV) to the ICV. The HRV was defined as the difference between the measured HV and the predicted HV. The predicted HV for each participant was calculated using the linear equation between HV and ICV, which was established by fitting a linear regression using AccuBrain®-measured HV and ICV data only from the control group (Supplementary Methods: Hippocampal residual volume calculation). The HVR and HRV both represent the hippocampal volume marker but are adjusted by the ICV-proportions method and the ICV-residuals approach, respectively (O’Brien et al., 2011).

### Data selection and exclusion

The scans that did not pass the quality control of the AccuBrain^®^ analysis, and the MMSE scores that did not have age recorded and matched scans were excluded from the analysis. Therefore, 678 scans from 66 participants (22 controls and 44 AD patients) were selected for the following statistical analysis, including a total of 179 pairs of back-to-back scans acquired on the same participants on the same day and 40 pairs of scans acquired at 2-week intervals. For further information on data inclusion and exclusion, please see Supplementary Methods: Data selection and exclusion.

### Statistical analysis

All statistical analyses were conducted in R [RStudio 4.0.5 (2021-03-31)], where a *p*-value < 0.05 was considered statistically significant. Independent *t*-tests were first used to compare the age, baseline HVR and baseline HRV between AD and controls, while Chi-squared test was used to compare sex distributions. For variables with skewed distributions (i.e., baseline MMSE, baseline AD-RAI and baseline QMTA), the non-parametric Mann-Whitney *U* test was used to enable group comparisons. For multiple comparisons of AD diagnosis between AD-RAI and HVR, HRV, or QMTA, the false discovery rate (FDR) was controlled below 0.05. The corresponding *q*-values were calculated and reported together with the original *p*-values before the FDR correction. A *q*-value < 0.05 was considered significant.

### Same-day repeatability and 2-week reproducibility of Alzheimer’s disease-resemblance atrophy index

The same-day repeatability of the AD-RAI was first examined. For individuals who received back-to-back MRI scans (*n* = 179), Bland-Altman plots (Bland and Altman, 1986) were used to visualise the association between the difference and average of the two AD-RAI scores calculated from these paired MRI scans. Pearson’s correlation was then used to quantify this association. Intraclass correlation coefficient (ICC) analysis (McGraw and Wong, 1996) was also employed to evaluate the agreement between the two AD-RAI scores generated from paired scans. For the ICC analysis, a two-way random effects model was used to take into account differences between participants as well as scanner fluctuations at different times. ICC less than 0.50, between 0.50 and 0.75, between 0.75 and 0.9, and greater than 0.9 indicates poor, moderate, good, and excellent agreement, respectively (Koo and Li, 2016).

Bland-Altman plots, Pearson’s correlation and ICC were also used to investigate 2-week reproducibility of AD-RAI, using 40 paired scans acquired at 2-week intervals for 40 participants. Two-week reproducibility was assessed as participants and scanner could undergo non-negligible fluctuations (Bartlett and Frost, 2008) unrelated to the brain pathological changes over time. We chose the 2-week interval under the assumption that the brain atrophy will not change significantly within 14 days.

### Discriminative ability of Alzheimer’s disease-resemblance atrophy index

We used logistic regression to examine whether baseline AD-RAI scores and MMSE-based classifications (i.e., AD: MMSE score ≤ 26; Control: MMSE score ≥ 27) predicted the probability of belonging to a given group (AD or control). The receiver operating characteristic (ROC) curve and the area under the curve (AUC) (Hanley and McNeil, 1982) were used to evaluate the discriminative ability of the logistic regression model. The optimal threshold range was identified by comparing predicted classifications with reference classifications (i.e., MMSE-based classifications) and calculating the confusion matrix including the true positive percentage (TPP, i.e., sensitivity) and the false percentage (FPP, i.e., 1-specificity). Separate logistic regressions were also run with single brain structural imaging markers (i.e., QMTA, HVR and HRV) as predictors of dementia status for comparison. The AUC between baseline AD-RAI and baseline QMTA, baseline HVR or baseline HRV was then statistically compared using DeLong’s test (DeLong et al., 1988).

### Longitudinal trajectories of Alzheimer’s disease-resemblance atrophy index

Linear mixed-effects (LME) models were used to examine longitudinal trajectories of AD-RAI in AD patients and controls (Fitzmaurice and Ravichandran, 2008). Using the R package nlme, the AD-RAI was modelled as a linear function of time, group and the interaction between time and group to examine (1) whether the average trajectories of AD-RAI in AD patients and controls significantly differ in their intercepts; (2) whether there is a constant rate of increase in the average AD-RAIs of all the participants for a single unit increase in time; and (3) whether the rates of the increase (the slopes of the average trajectories) over the 2 years are significantly different between AD and control groups. Further details on these analyses are provided in the Supplementary Methods: Linear mixed-effects models.

### Longitudinal trajectories of mini-mental state examination

To investigate whether baseline AD-RAI was associated with cognitive decline measured over a maximum of 2 years, we modelled the MMSE scores as a function of time, baseline AD-RAI and the interaction between time and baseline AD-RAI. More details are available in Supplementary Methods: Linear mixed-effects models.

To further assess the relationship between AD-RAI and MMSE, we used the repeated measures correlation (rmcorr) package (Bakdash and Marusich, 2017) to examine the longitudinal associations between repeated measures of AD-RAI and MMSE. This method assesses the common intra-individual variance and offers high statistical power to detect the common association between two measures, at the individual level. As AD patients had significantly higher baseline AD-RAI than the controls (Table 1) we expected the correlation between AD-RAI and MMSE to be different in AD patients compared to the controls. Therefore, we performed the rmcorr analysis for the two groups separately.

**TABLE 1:** Comparisons of age, sex, MMSE, AD-RAI, HVR, HRV, and QMTA at baseline across groups.

Variable	Normality test*	Controls	AD patients	Comparison
	(*P*-value)	(Mean ± SD**)	(Mean ± SD**)	(*P*-value)
Age	0.141	69.38 ± 7.21	69.13 ± 7.13	0.895^∧^
Female (%)	N/a	11 (50)	26 (59.09)	0.483^Δ^
MMSE	0.0002	30 (1)	19 (7)	3.87E-11^#^
AD-RAI	5.38E-10	0.083 (0.132)	0.997 (0.009)	2.20E-16^#^
HVR	0.479	0.0044 ± 0.0004	0.0037 ± 0.0005	5.82E-07^∧^
HRV (ml)	0.572	−0.00005 ± 0.51982	−1.128 ± 0.748	2.63E-08^∧^
QMTA	9.18E-07	0.362 (0.075)	0.792 (0.431)	7.49E-10^#^

*Shapiro-Wilk test; ^∧^Student’s t test; ^Δ^Chi-squared test; ^#^Mann-Whitney U test; **Normally distributed continuous variables were reported as Mean ± SD; while skewed continuous variables were reported as median (interquartile range) and categorical variables were reported as count (%). AD, Alzheimer’s disease; AD-RAI, Alzheimer’s disease-resemblance atrophy index; HVR, hippocampal volume ratio defined as the ratio of hippocampal volume to intracranial volume; HRV, hippocampal residual volume defined as the difference between the measured HV and the predicted HV; MMSE, Mini-Mental State Examination; QMTA, quantitative medial temporal lobe atrophy defined as the ratio of the inferior lateral ventricle to the ipsilateral hippocampal volume.

## Results

### Participant demographics

Table 1 summarises the demographics of participants included in the present analyses. In brief, participants with AD were on average 69.1 years old (± 7.13; 50% females) and controls were an average of 69.4 (± 7.21; 59.1% females). No significant differences in either age (*p* = 0.895) or sex (*p* = 0.483) between AD patients and controls. Baseline AD-RAI score were significantly higher in AD patients (Median AD-RAI = 0.997, Interquartile Range (IQR) = 0.009) relative to controls (Median AD-RAI = 0.083, IQR = 0.132; *p* = 2.20E-16), while baseline MMSE were significantly lower (median MMSE for AD patients = 19, IQR = 7; median MMSE for control patients = 30, IQR = 1; *p* = 3.87E-11). The baseline HVR and baseline QMTA were significantly lower and higher, respectively, in AD group than that in control group (*p* < 0.001). The baseline HRV was also significantly different between the control and AD group (*p* = 2.63E-08).

### Alzheimer’s disease-resemblance atrophy index had excellent same-day repeatability and 2-week reproducibility

**FIGURE 1:**
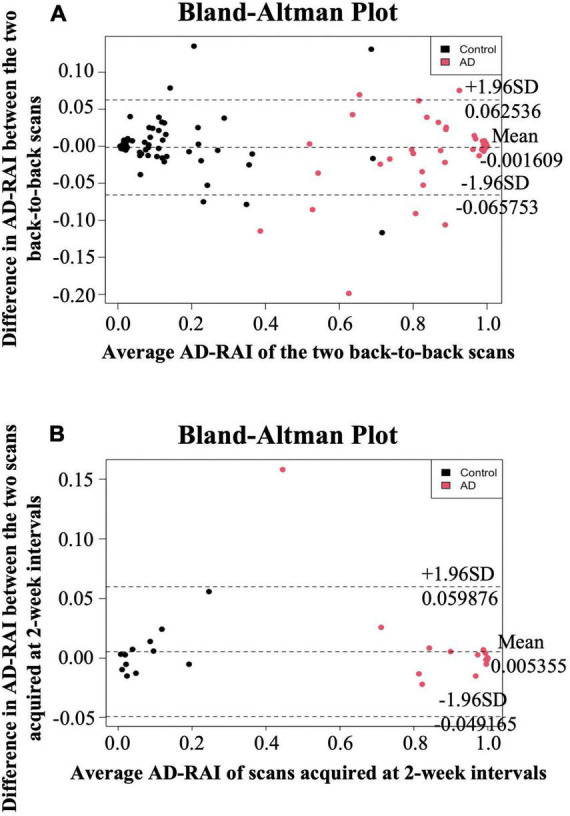
Bland-Altman plots for same-day repeatability and 2-week reproducibility analysis. The difference in AD-RAI of the paired scans [two back-to-back scans **(A)** or the two scans acquired at 2-week intervals **(B)**] was plotted against their average. The dashed lines in the middle, top and bottom indicate the mean difference, the mean difference plus or minus 1.96 times the standard deviation (SD) of the difference, respectively. AD-RAI, Alzheimer’s disease-resemblance atrophy index.

The mean difference in AD-RAI scores between the back-to-back scans was −0.002 [95% confidence interval (CI) = (−0.006 to 0.003)] (Table 2), which was not significantly different from zero (*p* = 0.512). Bland-Altman plots (Figure 1A) demonstrates the difference between the AD-RAI scores from back-to-back scans against their average, for each participant. No obvious trend were observed between the magnitude of the differences changes and size of the averages, and Pearson’s correlations between these metrics were not statistically significant (*p* = 0.801). ICC analyses also indicated a high level of agreement between the AD-RAI metrics (ICC = 0.997, 95% CI = [0.996, 0.998], *p* = 3.44E-198, Table 2). Taken together, the Bland-Altman method and the ICC method demonstrated the excellent same-day repeatability of AD-RAI.

**TABLE 2:** Same-day repeatability and 2-week reproducibility analysis results.

	Bland-Altman method					ICC method		
		
Analysis	Difference in AD-RAI		Pearson’s correlation coefficient			ICC	95%CI	*F*-test *P*-value
					
	Mean	95% CI	*r*	95% CI	*P*-value			
Same-day	−0.002	[−0.006, 0.003]	−0.019	[−0.165, 0.128]	0.801	0.997	[0.996, 0.998]	3.44E-198
2-week	0.005	[−0.003, 0.014]	−0.155	[−0.445, 0.165]	0.340	0.998	[0.996, 0.999]	1.06E-48

AD-RAI, Alzheimer’s disease-resemblance atrophy index; CI, confidence interval; ICC, intraclass correlation coefficient; r, Pearson’s correlation coefficient.

Due to the high agreement between AD-RAI scores of the back-to-back scans, AD-RAI computed from the first scan was used for all subsequent analyses. There was no statistically significant difference in AD-RAI scores between the paired scans 2-weeks apart (mean difference = 0.005, 95% CI = [−0.003 to 0.014], *p* = 0.231, Table 2). No significant correlation was found between the difference and the average in the corresponding Bland-Altman plot (*p* = 0.340, Table 2 and Figure 1B), and the ICC analysis showed an excellent agreement in AD-RAI scores between the paired scans (ICC = 0.998, 95% CI = [0.996, 0.999], *p* = 1.06E-48, Table 2), suggesting the excellent 2-week reproducibility of AD-RAI.

### Baseline Alzheimer’s disease-resemblance atrophy index scores have higher discriminative ability for Alzheimer’s disease relative to hippocampal volume ratio

**FIGURE 2:**
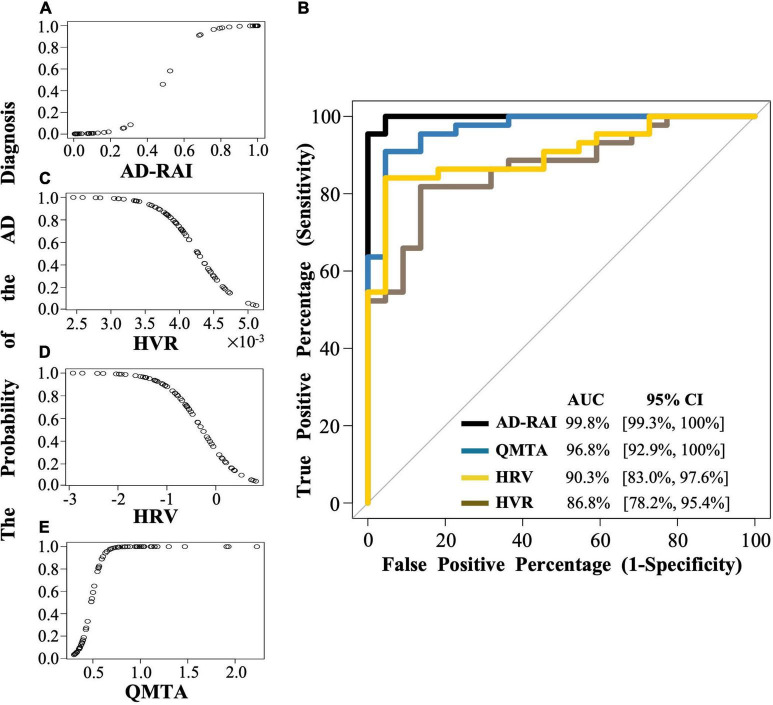
The logistic regression modelling and the ROC curves for AD-RAI, HVR, HRV and QMTA. **(A,C,D,E)** Each dot indicates the predicted probability of an AD diagnosis given baseline AD-RAI, HVR, HRV or QMTA. **(B)** The black line, brown line, yellow line, and blue line represent the ROC curve of AD-RAI, HVR, HRV and QMTA, respectively. The ROC curve was plotted with the true positive percentage (TPP) on *y*-axis against the false positive percentage (FPP) on *x*-axis, which represent the sensitivity and (1-specificity), respectively, at all the decision thresholds and were calculated based on the logistic regression modelling and the MMSE-based reference diagnosis. The diagonal line shows where the TPP is the same as the FPP. The AUC represent the area under the curve measuring the overall performance of the model to diagnose the AD. AD, Alzheimer’s disease; AD-RAI, Alzheimer’s disease-resemblance atrophy index; AUC, area under the curve; HVR, hippocampal volume ratio; HRV, hippocampal residual volume; QMTA, quantitative temporal lobe atrophy; ROC, receiver operating characteristic; MIRIAD, Minimal Interval Resonance Imaging in Alzheimer’s Disease.

A significant association was found between AD-RAI scores and the probability of belonging to AD/control group (Odds Ratio (OR) = 1.133, 95% CI = [1.044, 1.230], *p* = 0.003, Figure 2A and Table 3). Every increase of 0.01 in the AD-RAI increased the odds of belonging to AD group by 13.3% (Table 3). The AUC of this model was 99.8% (95% CI = [99.3%, 100%], Figure 2B black line). Table 4 demonstrates the sensitivity and specificity of the AD-RAI at different thresholds, where thresholding this metric between 0.46 and 0.58 resulted in a sensitivity between 95.45 and 100% and a specificity ranging from 95.45 to 100%.

**TABLE 3:** Results of the logistic regressions for AD-RAI, HVR, HRV and QMTA.

Predictor*	Odds ratio	95% Confidence interval of odds ratio		*P*-value
			
		Lower	Upper	
AD-RAI	1.133	1.044	1.230	0.003
HVR	0.687	0.567	0.833	0.0001
HRV	0.760	0.665	0.868	5.12E-05
QMTA	1.205	1.090	1.333	0.0003

*For a meaningful interpretation for the results of logistic regressions, we scaled up the AD-RAI, HVR, HRV and QMTA by 100, 10,000, 10 and 100 times, respectively, before performing the logistic regressions. AD, Alzheimer’s disease; AD-RAI, Alzheimer’s disease-resemblance atrophy index; HVR, hippocampal volume ratio defined as the ratio of hippocampal volume to intracranial volume; HRV, hippocampal residual volume defined as the difference between the measured HV and the predicted HV; QMTA, quantitative medial temporal lobe atrophy.

**TABLE 4:** Sensitivity, FPP and specificity at different decision thresholds of AD-RAI.

Thresholds		True positive percentage (TPP)	False positive percentage (FPP)	Specificity (%)
		Sensitivity (%)	1-specificity (%)	
			
AD-RAI	Probability of the AD diagnosis (P)			
0.409	0.072	100%	9.09%	90.91%
0.464	0.273	100%	4.55%	95.45%
0.501	0.520	97.73%	4.55%	95.45%
0.535	0.746	95.45%	4.55%	95.45%
0.579	0.913	95.45%	0	100%
0.593	0.940	93.18	0	100%

Logistic regression model formula: Log (P/1-P) = β_0_ + β_1_ × AD-RAI; β_0_ = −6.221, β_1_ = 12.500. AD, Alzheimer’s disease; AD-RAI, Alzheimer’s disease-resemblance atrophy index.

Logistic regression models including baseline HVR, baseline HRV and QMTA data (Figures 2C–E and Table 3) resulted in an AUC of 86.8% (95%CI = [78.2%, 95.4%]) for baseline HVR (Figure 2B brown line), an AUC of 90.3% (95%CI = [83.0%, 97.6]) for baseline HRV (Figure 2B yellow line) and an AUC of 96.8% (95%CI = [92.9%–100%]) for baseline QMTA (Figure 2B blue line). The AUC of the ROC curve of baseline AD-RAI (99.8%) was significantly higher than that of baseline HVR (DeLong’s test, *Z* = 2.98, *p*_–uncorrected_ = 0.003, *q* = 0.018) or baseline HRV (DeLong’s test, *Z* = 2.55, *p*_–uncorrected_ = 0.011, *q* = 0.033), while the difference in AUC of the ROC curves between baseline AD-RAI and baseline QMTA was not statistically significant as indicated by the DeLong’s test (*Z* = 1.66, *p*_–uncorrected_ = 0.098, *q* = 0.098). The results indicate that the performance of AD-RAI for AD diagnosis is equivalent to that of QMTA, and is better than that of HVR or HRV.

### Longitudinal trajectories of Alzheimer’s disease-resemblance atrophy index in Alzheimer’s disease patients and controls were distinct in the intercepts but not in the slopes

**FIGURE 3:**
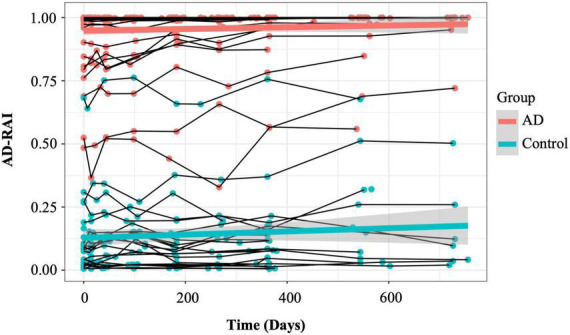
Longitudinal trajectories of AD-RAI in AD patients and controls. The thin lines connecting the dots represent the trajectories of AD-RAI of individual participants over time. The thick green and red lines represent the average trajectory of AD-RAI over time in control and AD groups, respectively. AD, Alzheimer’s disease; AD-RAI, Alzheimer’s disease-resemblance atrophy index.

Figure 3 demonstrates the longitudinal trajectories of AD-RAI scores in AD patients and controls during the 2-year follow-up time. The two average trajectories of AD-RAI for each group separated well from each other and both followed a slight upward pattern over the 2 year interval.

The LME results with AD-RAI as the outcome are presented in Table 5. Overall, there was a significant group effect on the AD-RAI (β = 0.81757, *p* < 0.0001), suggesting that the average baseline AD-RAI in AD patients was significantly higher than that in controls. However, there were no significant effect of time (*p* = 0.186, Table 5) or interaction between group and time (*p* = 0.353).

**TABLE 5:** Estimated coefficients based on LME model for the AD-RAI.

Fixed effects	Coefficients estimate			*t*-value	*P*-value
			
	Symbol	Estimate	Standard error		
Intercept	β_0_	0.12967	0.02862	4.531	<0.0001
Time	β_1_	0.00003	0.00002	1.325	0.186
Group (AD)	β_2_	0.81757	0.03505	23.327	<0.0001
Group × Time	β_3_	0.00025	0.00003	0.930	0.353

Model formula: AD-RAI_it_ = (β_0_ + b_0i_) + (β_1_ + b_1i_) × T_it_ + β_2_ × G_i_ + β_3_G_i_ × T_it_ + e_it_. AD-RAI, Alzheimer’s disease-resemblance atrophy index; LME, linear mixed-effects modelling.

### Changes in Alzheimer’s disease-resemblance atrophy index scores of Alzheimer’s disease patients are negatively associated with change in mini-mental state examination scores over time

Table 6 demonstrates the LME results where MMSE score was the outcome of interest. There was a significant effect of baseline AD-RAI on MMSE score (β = −10.8537, *p* < 0.0001) and a significant interaction between baseline AD-RAI and time (β = −0.0076, *p* = 0.0002), indicating higher baseline AD-RAI scores was associated with lower MMSE outcome and a steeper rate of change in MMSE over the 2-year observational window.

**TABLE 6:** Estimated coefficients based on LME Model for the MMSE.

Fixed effects	Coefficients estimate			*t*-value	*P*-value
			
	Symbol	Estimate	Standard error		
Intercept	β_0_	30.2874	0.8016	37.786	<0.0001
Time	β_1_	0.0002	0.0016	0.142	0.887
Baseline AD-RAI	β_2_	−10.8537	1.0169	−10.673	<0.0001
Baseline AD-RAI × Time	β_3_	−0.0076	0.0020	−3.744	0.0002

Model formula: MMSE_it_ = (β_0_ + b_0i_) + (β_1_ + b_1i_) × T_it_ + β_2_ × Baseline RAI_i_ + β_3_ Baseline RAI_i_ × T_it_ + e_it_. AD-RAI, Alzheimer’s disease-resemblance atrophy index; LME, linear mixed-effects modelling.

**FIGURE 4:**
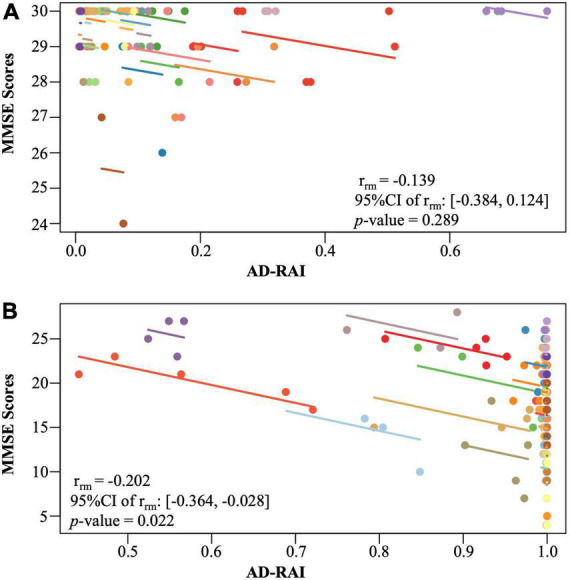
Intra-participant correlation between AD-RAI and MMSE in controls **(A)** and in AD patients **(B)**. The same-coloured dots represent paired measures of AD-RAI and MMSE taken on the same participant over the time. The coloured lines showed the correlation between AD-RAI and MMSE for each participant. AD-RAI, Alzheimer’s disease-resemblance atrophy index; MMSE, Mini-Mental State Examination.

No significant longitudinal association was found between changes in AD-RAI and changes in MMSE scores in the control group (*p* = 0.289, Figure 4A). However, a significant longitudinal negative association was found between AD-RAI scores and MMSE scores in AD group with the (*r*_*rm*_ = −0.202, 95% CI = [−0.364, −0.028], *p* = 0.022, Figure 4B).

## Discussion

In this study, we found that the AD-RAI had excellent same-day repeatability, 2-week reproducibility and discriminative ability. Repeatability and reproducibility are the most critical parameters for determining the reliability of any measurement tool. Excellent reproducibility over time is particularly important for an atrophy measuring tool intended to track disease progression to ensure that any detected changes are truly contributed by the changes of pathological atrophy. We also demonstrated that the AD-RAI predicted cognitive decline over a 2-year period and changes in the AD-RAI were sensitive to changes in MMSE scores.

Using MMSE-based classifications as reference categories, we found that the AD-RAI had an excellent discriminant ability for AD (AUC = 99.8%). We also found that when the AD-RAI was split to separate individuals at low/high risk, the thresholds between 0.46 and 0.58 resulted in the highest sensitivity (95.45 to 100%) and specificity (95.45 to 100%) relative to any other thresholds. Our result coincided with a previous study showing that 0.5 was the optimal AD-RAI threshold for differentiating between stable normal control (NC) and NC-to-MCI converters, as well as stable MCI from MCI-to-AD converters (Zhao et al., 2019). However, the range identified in our study is far from accurate and has limited clinical use in diagnosing AD. This is because there were very few data points with AD-RAI scores between 0.3 and 0.7 (Figure 2A), suggesting that this metric may not be accurate in predicting risk for individuals falling into this range. Larger studies with representative samples are therefore needed to provide more accurate estimates of the ideal diagnostic threshold to be used for the AD-RAI.

Compared to single region biomarkers, the AD-RAI (AUC = 99.8) had significant higher discriminative ability relative to the HVR (AUC = 86.8%) or HRV (AUC = 90.3), although performed equivalent to the QMTA (AUC = 96.8%). Our results are consistent with another recent study, where the AD-RAI performed better than HVR in participants whose reference diagnoses were based on amyloid β and tau pathology (Mai et al., 2021). In addition to HVR that normalised hippocampal volume by ICV-proportions method, we also applied the ICV-residuals method and computed the HRV to adjust for inter-participant variations in head size. A recent study showed that the ICV-residuals method performed better than other ICV-correction methods in neuroanatomical volume studies (Pintzka et al., 2015). Although the ICV-residuals method slightly improved the numerical value of the AUC of hippocampal volume marker (from 86.8 to 90.3%), that improvement was not statistically significant (DeLong’s test: *Z* = −1.76, *p*_–uncorrected_ = 0.078, *q* = 0.098), and the AD-RAI was significantly better in discriminative ability than the HRV. The hippocampus and other substructures within the medial temporal lobe represent the earliest affected regions in AD-related pathology (Scheltens et al., 2002; Frisoni et al., 2010), and hippocampal atrophy and MTA are well established as biomarkers for AD diagnosis at the MCI stage. However, a previous study revealed that ∼20% of amyloid biomarker-defined AD dementia patients do not show abnormalities with respect to hippocampal volume (Lowe et al., 2013), challenging the ability of the hippocampal volume as a marker in detecting hippocampal-sparing AD patients. While speculative, some of the patients in our sample may have fallen into the category of hippocampal-sparing AD, which could explain why the AD-RAI appeared to performed significantly better than the HVR or HRV in AD classification given its composite nature integrating atrophy a wide range of brain regions. We didn’t see significant differences in the performance of the AD-RAI relative to the QMTA as observed in Mai et al. (2021), where the amyloid β and tau pathology were included in the diagnostic standard. It is possible that our reference diagnostic criteria (i.e., MMSE only without pathologically confirmed diagnoses) may allow possible confounds leading to decreased statistical power in detecting a difference (to be discussed further later). We may test this possibility in future study in pathologically confirmed AD patients.

Our analyses indicated that higher baseline AD-RAI associated with a faster rate of decline in global cognition over time. A negative longitudinal correlation was also found between the AD-RAI and MMSE for AD patients only, but not in control participants. It has been well-established that the progress of whole brain atrophy was associated with the global cognitive decline in AD patients (Fox et al., 1999). Our study together with the previous study (Zhao et al., 2019) demonstrated that the AD-RAI successfully captured that association and can therefore be an effective marker for neurodegeneration in AD diagnosis defined by the 2018 NIA-AA research framework (Jack et al., 2018). With a tremendously growing number of prospective MRI studies of AD during the past decades, MRI-based automated computer classification of probable AD versus controls as well as disease prediction using various machine learning and pattern recognition techniques have been an intensive focus in AD research (Frizzell et al., 2022), leading to the development of many synthetic atrophy indices, such as SPARE-AD index, AD-PS scores, STAND scores, etc. (Davatzikos et al., 2009; Misra et al., 2009; Vemuri et al., 2009; Spulber et al., 2013; Casanova et al., 2018). AD-RAI is conceptually similar to but methodologically different from those indices. Being a strong predictor of the clinical cognitive decline in AD patients as evidenced by our study, the AD-RAI provides a promising option in a pool of available auxiliary diagnosis tools for AD, which is objective, time-saving, clinically meaningful and easy to understand, and may play an important role in the detection and management of AD.

### Strengths and limitations

The strengths of this study were that the MIRIAD dataset offered data quality ideal for methods validation. Both control and AD participants received up to nine serial scans from entering the cohorts to 2 years with a wide range of time intervals and with back-to-back scans within 1 day at three time points for most of the participants, and all scans were conducted on the same scanner and acquired by the same radiographer, eliminating the variability from different machines and different radiographers. Those features allowed sufficient numbers of paired scans and statistical power to assess the same-day repeatability and 2-week reproducibility, where we specifically tested the agreement between the paired AD-RAI measurements by choosing the Bland-Altman method (Bland and Altman, 1986) and the ICC method (McGraw and Wong, 1996) rather than a paired *t*-test or a sole Pearson’s correlation test. The former could not distinguish the true equality from the situation that the difference between the paired measurements relates to the value of their average measurement; and the later could only assess the degree of the association between the paired measurements but not their equality (Berchtold, 2016). In addition, we chose rmcorr to detect the common longitudinal association between AD-RAI and MMSE from the longitudinally paired repeated measures of each participant, which provides greater statistical power and is more suitable than the popular Pearson correlation method that needs to average the repeated data for each participant before performing the correlation and thus could only assess the inter-individual correlation with the data’s longitudinal feature lost (Bakdash and Marusich, 2017).

However, this study also has several limitations. After data exclusion, only 22 control and 44 AD participants were included in the analysis. The small sample size likely reduced the statistical power of our trajectory study. For example, we did not find a significant slope in AD-RAI trajectories in either AD or the control group during the 2-year follow-up time, which might be because the power of the statistical test was too low to detect the small but real change in brain atrophy within the relatively short period. An alternative explanation for the lack of significant slopes in AD-RAI is possible ceiling effects. By carefully examining the data, we found that 35 participants among the 44 AD patients had an AD-RAI value greater than 0.9 (where the maximum value is 1). It is possible that the rate of brain volume loss may slow down when the global atrophy has reached a certain severity level. In that case, the average change in AD-RAI value could be hard to detect when the data contained large numbers of AD participants with very high AD-RAI scores. The imbalanced distribution of AD-RAI in the participants in MIRIAD dataset also raises caution in explaining the optimal threshold range (0.46 to 0.58). Because of the imbalanced distribution of AD-RAI in our sample (i.e., very few participants with AD-RAI scores between 0.3 and 0.7), our findings may not generalise cohorts with more heterogeneous AD-RAI scores. For example, our study could not address whether AD-RAI can be used for differentiating between preclinical AD and healthy controls and for predicting their disease progression. Overall, replication studies with larger sample size and a greater diversity of patients in the different stages of AD (e.g., amnestic MCI) will help to address the discussed shortcomings of this present study.

The MIRIAD dataset based its diagnosis of probable AD on the NINCDS-ADRDA clinical Criteria (McKhann et al., 1984), due to the lack of biomarker data (e.g., amyloid β and pathological tau) available for a pathologically confirmed diagnosis. Hence, we are unable to rule out the possibility that our control group contained participants with cognitively unimpaired preclinical AD and that the AD group could contain participants who may have neurodegenerative disorders that closely resemble AD symptomology (Lombardi et al., 2020). This limitation represents a potential confound in our data and as such, weakens the conclusion we could draw from our analyses. This limitation may also explain why we did not observe any significant difference in the AUC between AD-RAI and QMTA.

Alzheimer’s disease diagnosis in clinic is often challenged by the facts that (1) significant heterogeneity of regional brain atrophy patterns occurs in AD (Dong et al., 2017; Risacher et al., 2017); (2) other neurodegenerative disorders, such as vascular dementia and Lewy’s body dementia, often coexist with AD (Kling et al., 2013; Davey, 2014); and (3) AD may overlap with other neurodegenerative diseases in brain regions undergoing neurodegeneration, such as Parkinson’s (Weintraub et al., 2012). Neither our study nor several previous studies (Zhao et al., 2019; Liu et al., 2021; Mai et al., 2021) on AD-RAI included patients with other pathologically confirmed neurodegenerative disorders. Hence, the specificity of AD-RAI in AD diagnosis remains to be tested in real-world scenarios. A recent study (Yu et al., 2021) showed that the AD-RAI alone could not distinguish between AD and FTD. However, a combination of AD-RAI with another newly developed Frontotemporal dementia (FTD) index, and with a sequential decision strategy offered a solution to differentiate AD and FTD (Yu et al., 2021). Future studies on mixed groups of different pathologically confirmed neurodegenerative disorders or on patients with brain atrophy contributed simultaneously by two or more types of pathological processes, could further improve the performance of AD-RAI and fine-tune its application.

Early diagnosis of AD by using structural MRI markers is also hindered by the reality that brain atrophy is an outcome of pathological changes at molecular and cellular levels that precede detectable anatomic changes. Previous study found that the sensitivity of AD-RAI was lower than that of hippocampal volume in detecting preclinical AD (Liu et al., 2021). Therefore, how early the AD-RAI could capture the macroscopic brain alterations in association with the molecular-level pathological changes of AD, and whether regional volumetric features could be taken into consideration together with the whole brain atrophic pattern to improve the sensitivity in early AD diagnosis is also an interesting topic to explore.

## Conclusion

In conclusion, we found that the AD-RAI had excellent repeatability, reproducibility and discriminative ability in AD and demonstrated that longitudinal changes in this neuroimaging-derived metric were sensitive to cognitive decline in AD patients. Our findings suggest that the AD-RAI represents a promising biomarker that could aid earlier detection of participants at risk of developing AD allowing for earlier intervention and clinical trial recruitment.

## Data availability statement

The dataset supporting the conclusions of this article is available in the MIRIAD XNAT database. The MRI scans and the associated demographic data and MMSE scores can be downloaded in the NIFITI (Neuroimaging Informatics Technology Initiative) format and as CSV files, respectively.

## Ethics statement

The following statement can be found in the article that released the MIRIAD dataset: Ethical approval for the study (and subsequently its release) was received from the local research ethics committee, and written consent obtained from all participants (Malone et al., 2013).

## Author contributions

QH analysed and interpreted the data, prepared the manuscript, and including all tables and figures. LS and YL processed the MIRIAD dataset on the AccuBrain^®^ system to generate AD-RAI. LS, CW, and IM provided technical support and feedback on the manuscript. VM initiated the study. JC and MA provided essential guidance in study design, data analysis, and data interpretation. MA provided guidance on all versions of the manuscript. All authors contributed to the article and approved the final manuscript.
