# Supplementary material for: Validation of the Alzheimer’s disease-resemblance atrophy index in classifying and predicting progression in Alzheimer’s disease
Source: Front Aging Neurosci. 2022 Aug 5;14:932125. doi: 10.3389/fnagi.2022.932125 (PMC9435378; doi:10.3389/fnagi.2022.932125)
Supplement: Supplementary file 1 [file Data_Sheet_1.docx]

**Supplementary Methods**

**Image acquisition and preprocessing**

T1-weighted images were acquired for the 69 participants multiple times using an inversion recovery prepared fast spoiled gradient recalled (IR-FSPGR) sequence on a 1.5 T Signa MRI scanner (GE Medical Systems, Milwaukee,WI, USA) by the same MRI radiographer and with the following parameters: field of view 24 cm, 256 $\times$256 matrices, 124 1.5mm coronal partitions, TR 15ms, TE 5.4ms, flip angle 15◦, and TI 650ms. A total of 708 scans were obtained over up to 2 years of follow-up. Among the 708 scans, 185 pairs of scans (back-to-back scans within one day) were acquired at different time points for 185 participants (Supplemental Table 1). From the 708 scans, 42 pairs of scans that were acquired at 2-week (defined as 14.6 days based on the calculation from the recorded ages) intervals for the 42 participants can be extracted (Supplemental Table 2). About every 6 months (26 weeks), the participants also received MMSE re-evaluation (Supplemental Fig. 1).

Images first underwent a series of preprocessing steps, including noise reduction, bias field correction, and intensity normalization. Processed images were then compared and matched with pre-stored images in the atlas of AccuBrain®. After the registration, the pre-encoded, radiologist-defined-brain parcellation labels of those highly similar pre-stored images are transformed to the participant images and fused to produce the volumetric segmentation results. The quantification of brain volumetry was performed on selected cognitive-relevant regions including brain parenchyma, typical subcortical structures (bilateral hippocampus and amygdala), ventricular regions (ventricular system, lateral ventricle, inferior lateral ventricle), and lobar regions (frontal lobe, occipital lobe, temporal lobe, parietal lobe, cingulate lobe and insular).

**Data selection and exclusion**

A flowchart of data selection and exclusion can be found in Supplemental Fig. 2. Among the 708 scans, 26 scans did not pass the quality control (QC) of AccuBrain® analysis (Supplemental Table 3). Among those 26 scans, 7 scans had the problem of low contrast and were excluded from the analysis (but the related participants and their other data were still included in the study); another 7 scans from a male participant had position error, and that male participant only had those 7 scans, thus he and all his data were consequently excluded from the analyses; the left 12 scans showed severe atrophy which exceeded the analysis capacity of AccuBrain®. Interestingly, those 12 scans belonged to a 75.82-year-old male participant who was assigned in the control group with a stable MMSE score of 30 all the time. That male participant was also excluded from the analysis. In addition, a female participant did not have baseline age and baseline scan available. So, she was excluded from the analysis as well where the baseline age and baseline scan information were required. The original numbers of participants and scans as well as the numbers after exclusion were summarized in Supplemental Table 4. After the exclusion of the 26 scans that did not pass the QC, 179 pairs of back-to-back scans instead of 185 pairs (Supplemental Table 1), and 40 pairs of scans acquired at 2-week intervals instead of 42 pairs (Supplemental Table 2) were used later for the same-day repeatability assessment and for the intra-time reproducibility assessment respectively. When we combined the participants’ scan information and clinical assessment (MMSE) downloaded from the [MIRIAD XNAT database](http://miriad.drc.ion.ucl.ac.uk/atrophychallenge) together, we found that there was no scan available to match several MMSE scores of 3 participants (Supplemental Table 5). The ages when those MMSE scores were recorded were not available either. Therefore, those MMSE scores were excluded for MMSE-related analysis.

**Hippocampal residual volume calculation**

The following linear regression between hippocampal volume (HV) and intracranial volume (ICV) was fitted using HV and ICV data of the control participants only. Both HV and ICV were obtained automatically through AccuBrain® and were designated as HV_-measured_ and ICV_-measured_ respectively.

**HV_-predicted_ = Coefficient_-intercept_ + Coefficient_-ICV_ × ICV_-measured_**

The following parameters in the above model (multiple R-squared = 0.2801; adjusted R-squared = 0.244) were obtained:

**Coefficient_-intercept_** = 2.6655388 (95%CI = [-0.3194645784, 5.650542098]; *p*-value = 0.0773)

**Coefficient_-ICV-measured_** = 0.0026323 (95%CI = [0.0006637552, 0.004600781]; *p*-value = 0.0113).

The hippocampal residual volume (HRV) was then calculated for each participant in either control or AD group using the following formula.

**HRV = HV_-measured_ - HV_-predicted_ = HV_-measured_ -2.6655388 - 0.0026323ICV_-measured_**

**Linear mixed-effects models**

***Trajectories of AD-RAI***

The following formula was used to model AD-RAI as a function of time, group and the interaction between time and group.

**AD-RAI_𝑖𝑡_ = (𝛽_0_ + b_0𝑖_) + (𝛽_1_ + b_1𝑖_) ×T_𝑖𝑡_ + 𝛽_2_ ×G_𝑖_ + 𝛽_3_G_𝑖_ × T_𝑖𝑡_ + 𝑒_𝑖𝑡_**

In the model: **𝑖** = participant ID; **𝑡** = time point; **AD-RAI_𝑖𝑡_** is a continuous variable representing the AD-RAI measure on **𝑖^th^** participant at time **𝑡**; **𝛽_0_** represents the population-averaged intercept i.e., the average AD-RAI of all the participants without the effect from the group at the beginning of the study; **b_0𝑖_** represents the random intercept for **𝑖^th^** participant which is the variance calculated from the measure of the difference between the **𝑖^th^** participant’s intercept (individual AD-RAI) and the population-averaged intercept (the average AD-RAI of all the participants without the effect from the group) at the beginning of the study; **T_𝑖𝑡_** is a continuous variable representing the timing of the AD-RAI measure on the **𝑖^th^** participant at time **𝑡**; **𝛽_1_** is the coefficient for time measuring the population-averaged slope over time, which, in our study, indicates the constant rate of change in the average AD-RAI of all the participants for a single unit increase in time; **b_1𝑖_** represents the random slope for **𝑖^th^** participant, which is the variance calculated from the measure of the difference between the **𝑖^th^** participant’s slope (changing rate of individual AD-RAI over time) and the population-averaged slope (change rate of the average AD-RAI of all the participants over time without the effect from the group); **G_𝑖_** represents the group category of the **𝑖^th^** participant, which is a categorical variable containing two values, 0 for controls and 1 for AD patients in our study; **𝛽_2_** is the coefficient for group measuring the difference in the population-averaged intercepts i.e., the average AD-RAI, between AD patients and controls at the beginning of the study; **G_𝑖_ × T_𝑖𝑡_** represents the interaction between group and time; **𝛽_3_** is the coefficient for the interaction of group and time measuring the difference in the population-averaged slopes over time between AD patients and controls, which can be used to answer the question of whether the slopes of the longitudinal trajectories of AD-RAI between AD patients and controls are the same; **𝑒_𝑖𝑡_** represents the random error.

***Prediction of Cognitive Decline***

**MMSE_𝑖𝑡_ = (𝛽_0_ + b_0𝑖_) + (𝛽_1_ + b_1𝑖_) ×T_𝑖𝑡_ + 𝛽_2_ ×BaselineRAI_𝑖_  + 𝛽_3_BaselineRAI_𝑖_ × T_𝑖𝑡_ + 𝑒_𝑖𝑡_**

In the model: **𝑖** = participant ID; **𝑡** = time point; **MMSE_𝑖𝑡_** represents the MMSE record on **𝑖^th^** participant at time **𝑡** and is treated as a continuous variable; **𝛽_0_** represents the population-averaged intercept i.e., the average MMSE score for people without atrophy at the beginning of the study; **b_0𝑖_** represents the random intercept for **𝑖^th^** participant, which is the variance calculated from the measure of the difference between the **𝑖^th^** participant’s intercept (individual MMSE score) and the population-averaged intercept (the average MMSE score without atrophy) at the beginning of the study; **T_𝑖𝑡_** is a continuous variable representing the timing of the MMSE record on the **𝑖^th^** participant at time **𝑡**; **𝛽_1_** is the coefficient for time measuring the population-averaged slope over time, which, in our study, indicates the constant rate of change in the average MMSE score without atrophy for a single unit increase in time; **b_1𝑖_** represents the random slope for **𝑖^th^** participant, which is the variance calculated from the measure of the difference between the **𝑖^th^** participant’s slope (changing rate of individual MMSE score over time) and the population-averaged slope (change rate of average MMSE score over time without atrophy); **BaselineRAI_𝑖_** represents the AD-RAI measure on **𝑖^th^** participant at the beginning of the study, indicating the atrophy level of that participant; **𝛽_2_** is the coefficient for BaselineRAI_𝑖_ measuring the constant rate of change in the average MMSE score for a single unit increase in baseline AD-RAI; **BaselineRAI_𝑖_ × T_𝑖𝑡_** represents the interaction between baseline AD-RAI and time; **𝛽_3_** is the coefficient for the interaction of baseline AD-RAI and time measuring the rate of change in the population-averaged slopes with time for different baseline AD-RAI, which can be used to answer the question of whether the AD-RAI measured at the initial visit can predict the speed of the clinical cognitive decline; **𝑒_𝑖𝑡_** represents the random error.

**Supplementary Figures**

**
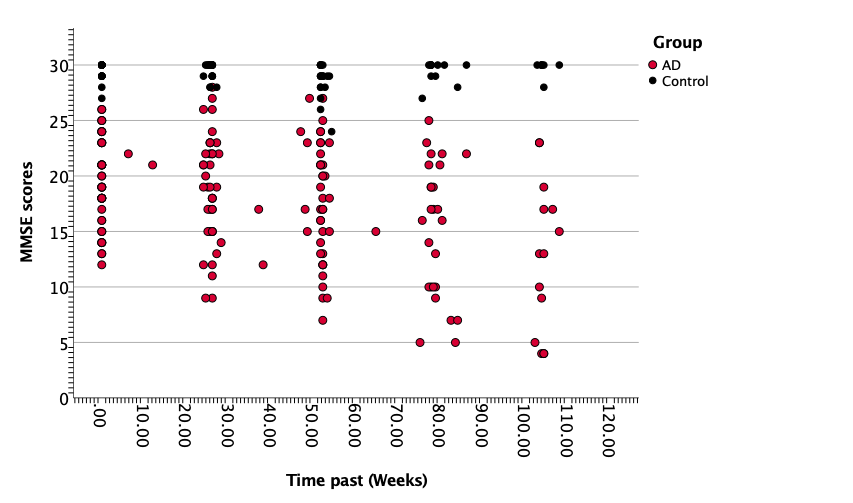
**

**Supplemental Fig. 1**: Scatter plot of MMSE scores versus the time. The figures show that the MMSE scores for each participant in the cohorts were updated about every 26 weeks.


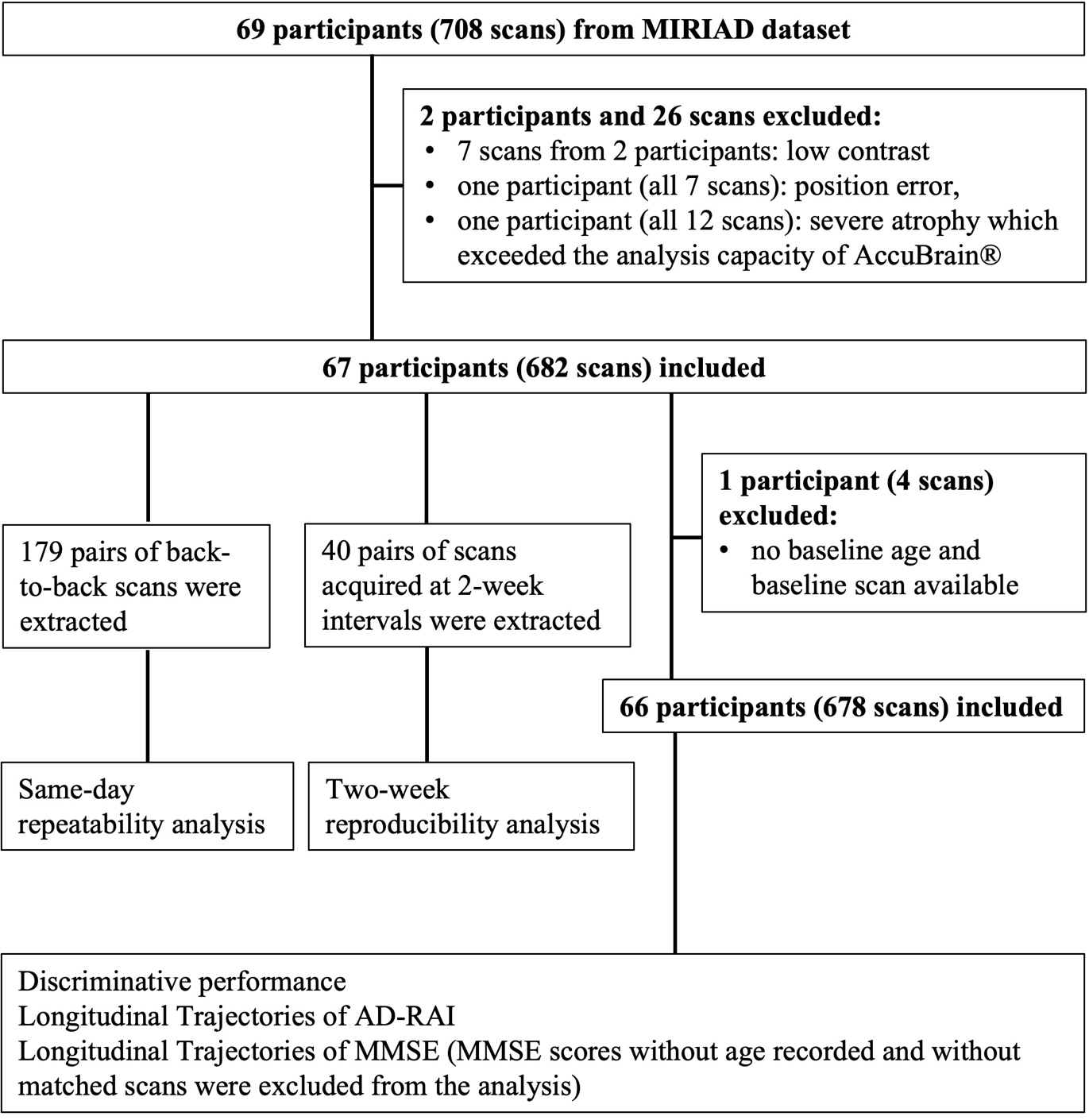


**Supplemental Fig. 2**: Flowchart of data selection and exclusion.

**Supplemental Tables**

**Supplemental Table 1**: Number of participants receiving back-to-back scans at indicated time points

| **Time past (Weeks)** | **Number of participants (scans)** | | **Total pairs of scans** |
| --- | --- | --- | --- |
|  | **AD** | **Control** |  |
| 0.00 | 43 (86) | 22(44)/**20(41)*** | 185/**179*** |
| 4.17 | 1(2) | 0(0) |  |
| 4.69 | 2(4) | 1(2) |  |
| 5.21 | 2(4) | 1(2) |  |
| 5.74 | 13(26) | 8(16)/**7(14)*** |  |
| 6.26 | 13(26) | 6(12)/**5(10)*** |  |
| 6.78 | 5(10) | 4(8) |  |
| 7.30 | 3(6) | 1(1) |  |
| 7.82 | 1(2) | 0(0) |  |
| 8.34 | 1(2) | 0(0) |  |
| 8.86 | 0(0) | 1(2) |  |
| 32.85 | 1(2) | 1(2) |  |
| 33.89 | 1(2) | 0(0) |  |
| 34.94 | 0(0) | 1(2) |  |
| 35.98 | 1(2) | 1(2) |  |
| 37.02 | 1(2) | 0(0) |  |
| 37.54 | 4(8) | 4(8) |  |
| 38.06 | 18(36) | 7(14)/**6(12) *** |  |
| 38.59 | 1(2) | 2(4) |  |
| 39.11 | 2(4) | 2(4)/**1(2) *** |  |
| 39.63 | 1(2) | 0(0) |  |
| 40.15 | 1(2) | 1(2) |  |
| 40.67 | 1(2) | 0(0) |  |
| 41.19 | 1(2) | 0(0) |  |
| 41.71 | 1(2) | 0(0) |  |
| 43.80 | 1(2) | 1(2) |  |
| 46.41 | 0(0) | 1(2) |  |
| 48.49 | 0(0) | 1(2) |  |

*11 scans in 6 pairs of back-to-back scans, all from control group did not pass the quality control of the AccuBrain® analysis. So, only 179 pairs of back-to-back were used later for the same-day repeatability assessment.

**Supplemental Table 2**: Pairs of the scans acquired at two-week intervals

| **Time interval (days)** | **Pairs of scans** | | **Total pairs of scans** |
| --- | --- | --- | --- |
|  | **AD** | **Control** |  |
| 14.6 | 29/**28*** | 13/**12*** | 42/**40*** |

*2 pairs of scans, one from probable AD group and one from control group did not pass the quality control of the AccuBrain® analysis. So, only 40 pairs scans were used later for the two-week reproducibility assessment.

**Supplemental Table 3**: 26 scans shown in red did not pass the QC of Accubrain®.

| **miriad_219** | **miriad_223** | **miriad_240** | **miriad_241** |
| --- | --- | --- | --- |
| miriad_219_1_MR_1  (Low contrast) | miriad_223_1_MR_1  (Severe atrophy) | miriad_240_1_MR_1  (Position error) | miriad_241_1_MR_1 |
| miriad_219_1_MR_2 | miriad_223_1_MR_2  (Severe atrophy) | miriad_240_2_MR_1  (Position error) | miriad_241_1_MR_2 |
| miriad_219_3_MR_1  (Low contrast) | miriad_223_2_MR_1  (Severe atrophy) | miriad_240_3_MR_1  (Position error) | miriad_241_2_MR_1 |
| miriad_219_3_MR_2  (Low contrast) | miriad_223_3_MR_1  (Severe atrophy) | miriad_240_4_MR_1  (Position error) | miriad_241_3_MR_1 |
| miriad_219_4_MR_1 | miriad_223_3_MR_2  (Severe atrophy) | miriad_240_5_MR_1  (Position error) | miriad_241_3_MR_2 |
| miriad_219_5_MR_1 | miriad_223_4_MR_1  (Severe atrophy) | miriad_240_6_MR_1  (Position error) | miriad_241_4_MR_1 |
| miriad_219_6_MR_1  (Low contrast) | miriad_223_5_MR_1  (Severe atrophy) | miriad_240_7_MR_1  (Position error) | miriad_241_5_MR_1 |
| miriad_219_6_MR_2  (Low contrast) | miriad_223_6_MR_1  (Severe atrophy) |  | miriad_241_6_MR_1 |
| miriad_219_7_MR_1  (Low contrast) | miriad_223_6_MR_2  (Severe atrophy) |  | miriad_241_6_MR_2 |
|  | miriad_223_7_MR_1  (Severe atrophy) |  | miriad_241_7_MR_1  (Low contrast) |
|  | miriad_223_9_MR_1  (Severe atrophy) |  | miriad_241_9_MR_1 |
|  | miriad_223_10_MR_1  (Severe atrophy) |  |  |

**Supplemental Table 4**: Number of participants and scans

|  | **Original Data from MIRIAD** | |  | **Data for Analysis after exclusion*** | |
| --- | --- | --- | --- | --- | --- |
|  | **No. of Participants** | **No. of Scans** |  | **No. of Participants** | **No. of Scans** |
| AD | 46 | 465 |  | 44 | 454 |
| Control | 23 | 243 |  | 22 | 224 |
| Total | 69 | 708 |  | 66 | 678 |

*The participant **miriad_223 (control)** and participant **miriad_240 (AD)** were excluded due to that none of the scans of those two participants passed the quality control of the AccuBrain® analysis. The participant **miriad_256 (AD)** was excluded due to no baseline age and baseline scan were available. Another 7 scans were also excluded for the same reason.

**Supplemental Table 5**: MMSE scores without matched scans and ages

| **Participant** | **Scan ID** | **EXT_CLINICALASSESSMENT**  **DATA ID** | **MMSE** | **sumbox** | **rating** | **Age** | **Gender** | **Group** |
| --- | --- | --- | --- | --- | --- | --- | --- | --- |
| miriad_219 | miriad_219_1_MR_1 | miriad_219_1_MMSE | 28 |  |  | 76.52 | Male | Control  Control  Control  Control  Control  Control  Control  Control  Control  Control |
|  | miriad_219_1_MR_2 |  |  |  |  | 76.52 |  |  |
|  | miriad_219_3_MR_1 |  |  |  |  | 76.64 |  |  |
|  | miriad_219_3_MR_2 |  |  |  |  | 76.64 |  |  |
|  | miriad_219_4_MR_1 |  |  |  |  | 76.79 |  |  |
|  | miriad_219_5_MR_1 | miriad_219_5_MMSE | 29 |  |  | 77.02 |  |  |
|  | miriad_219_6_MR_1 | miriad_219_6_MMSE | 30 |  |  | 77.25 |  |  |
|  | miriad_219_6_MR_2 |  |  |  |  |  |  |  |
|  | miriad_219_7_MR_1 |  |  |  |  | 77.52 |  |  |
|  | **?** | **miriad_219_8_MMSE** | **29** |  |  | **?** |  |  |
| miriad_253 | miriad_253_1_MR_1 | miriad_253_1_MMSE | 18 | 9 | 2 | 74.59 | Female | AD |
|  | miriad_253_1_MR_2 |  |  |  |  | 74.59 |  |  |
|  | miriad_253_2_MR_1 |  |  |  |  | 74.63 |  |  |
|  | miriad_253_3_MR_1 |  |  |  |  | 74.69 |  |  |
|  | miriad_253_3_MR_2 |  |  |  |  | 74.69 |  |  |
|  | miriad_253_4_MR_1 |  |  |  |  | 74.86 |  |  |
|  | miriad_253_5_MR_1 | miriad_253_5_MMSE | 19 |  |  | 75.05 |  |  |
|  | miriad_253_6_MR_1 |  |  |  |  | 75.32 |  |  |
|  | miriad_253_6_MR_2 |  |  |  |  | 75.32 |  |  |
|  | miriad_253_7_MR_1 | miriad_253_7_MMSE | 12 |  |  | 75.59 |  |  |
|  | **?** | **miriad_253_8_MMSE** | **14** |  |  | **?** |  |  |
| miriad_256 | **?** | **miriad_256_1_MMSE** | **16** | **10** | **2** | **?** | Female | AD |
|  | miriad_256_3_MR_1 |  |  |  |  | 76.53 |  |  |
|  | miriad_256_4_MR_1 |  |  |  |  | 76.68 |  |  |
|  | miriad_256_5_MR_1 | miriad_256_5_MMSE | 14 |  |  | 76.91 |  |  |
|  | **?** | **miriad_256_6_MMSE** | 12 |  |  | **?** |  |  |
|  | miriad_256_7_MR_1 |  |  |  |  | 77.41 |  |  |
|  | **?** | **miriad_256_8_MMSE** | **7** |  |  | **?** |  |  |
